# Screening of Graves' disease susceptibility genes by whole exome sequencing in a three-generation family

**DOI:** 10.1186/s12920-020-00865-z

**Published:** 2021-02-10

**Authors:** Zhuoqing Hu, Wei Li, Miaosheng Li, Hao Wei, Zhihui Hu, Yanting Chen, Ai Luo, Wangen Li

**Affiliations:** 1 Department of Endocrinology, The Second Affiliated Hospital of Guangzhou Medical University, Guangzhou, 510220 China; 2 Huizhou Health Sciences Polytechnic, Huizhou, China; 3 Department of Endocrinology, Affiliated Hospital of Guangdong Medical University, Zhanjiang, China

**Keywords:** Graves’ disease, Susceptibility genes, Three generations, Whole exome Sequencing

## Abstract

**Background:**

Graves’ disease(GD) has a tendency for familial aggregation, but it is uncommon to occur in more than two generations. However, little is known about susceptibility genes for GD in the three-generation family.

**Methods:**

DNA were extracted from three-generation familial GD patient with a strong genetic background in a Chinese Han population. The Whole Exome Sequencing (WES) was utilized to screen the genome for SNVs associated with GD and the Sanger Sequencing was used to confirm the potential disease-causing genes.

**Results:**

In the case study, there were five patients with Graves’ disease(GD) from a three-generation family. The SNVs of *MAP7D2*(c. 452C > T: p. A151V), *SLC1A7*(c. 1204C > T: p. R402C), *TRAF3IP3*(c. 209A > T: p. N70I), *PTPRB*(c. 3472A > G: p. S1158G), *PIK3R3*(c. 121C > T: p. P41S), *DISC1*(c. 1591G > C: p. G531R) were found to be associated with the familial GD and the Sanger sequencing had confirmed these variations. Furthermore, PolyPhen-2 score showed that the variants in *TRAF3IP3*, *PTPRB*, *PIK3R3* are more likely to change protein functions.

**Conclusion:**

The *MAP7D2*, *SLC1A7*, *TRAF3IP3*, *PTPRB*, *PIK3R3*, *DISC1* may be the candidate susceptibility genes for familial GD from a three generations family.

## Background

Graves’ disease is a thyroid-related autoimmune disorder which is caused by a complex interaction between susceptibility genes and multiple environmental factors. Previous familial and twin studies had shown that there was an association of genetic factors with Graves’ disease in 79% cases of Graves’ Disease which influenced the familial clustering in GD [1]. People with other autoimmune diseases such as type 1 diabetes or rheumatoid arthritis are also more likely to suffer from this disease. In addition, past reports reported that smoking increases the chance of Graves’ Disease. Other causes of Graves’ Disease may also include stress, infection, or childbirth. The prevalence of overt hyperthyroidism ranges from 0.2% to 1.3% in in the general population [2]. In China, the prevalence of hyperthyroidism is about 1.3% [3]. Graves’ disease is a multisystem syndrome including hypermetabolic syndrome, diffuse goiter, eye signs, skin lesions, and thyroid acropathy. The basic treatments of Graves’ disease are antithyroid drug treatment, radionuclide iodine treatment, surgical treatment and interventional embolization treatment. Family linkage analysis, candidate gene method and genome-wide association analysis (GWAS) have identified a greater number of Graves’ disease susceptibility loci as well. In GWAS, the existing sequence variations are identified from the whole human genome and the variations that related to the disease are screen out. GWAS method has allowed many previously undiscovered genes and chromosomal regions to be detected which help provide many clues to the pathogenesis of complex diseases. However, all the variants that have been discovered have little to the heritability of GD. Therefore, different approaches were applied in this case study to identify the more susceptibility loci.

In addition to twins study, the familial GD is the ideal object of study on contribution of genetic heritability to complex disease. Findings from family linkage analysis indicated that the 5q31-q33, 6p, 7q, 8q, 10q, 12q, 14q and 20q regions were related to GD susceptibility [4–6]. Subsequent research had verified that there was a linkage of familial GD to *HLA* gene and *CTLA-4* gene. However, linkage analysis has not addressed the need for fine gene mapping in complex diseases and linkage analysis also requires a large familial sample size to localize the pathogenic genes by observing whether the genetic markers are co-segregated with the disease. In contrast, Whole Exome Sequencing can explore the susceptibility genes with a small sample size. Whole Exome Sequencing is an efficient strategy for fine mapping to determine the exact location of variant [7, 8]. In the case study, WES was utilized to screen disease-causing genes from a three-generation familial GD patient with a strong genetic background of Chinese Han.

## Methods

### Study participants

**Fig. 1:**
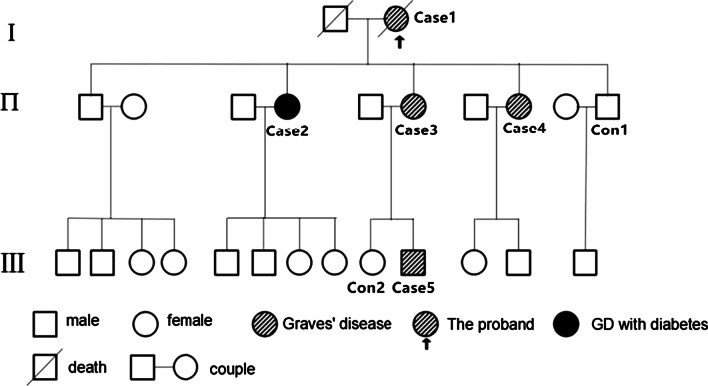
Pedgree of study family

A three-generation family from Zhanjiang, Guangdong Province had been targeted for the case study. There were 25 people in the family tree including 13 males and 12 females (Fig. 1.). Five members were diagnosed with GD (Case 2 had type 2 diabetes simultaneously). Two healthy people in the first-degree relatives of the family were set as control group at the same time. Both the cases and control group were confirmed based upon medical history, physical examination, and results of thyroid function examination. All the five GD patients had typical clinical manifestation of hyperthyroidism such as heart and hands trembling. Four of the patients resorted to drug (Methimazole) for anti-thyroid treatment and one other patient resorted to I^131^ for anti-thyroid treatment. As of the month before the article was submitted, it was confirmed that the control group was still free of Graves' disease. Table 1 has the details of the participants.

**Table 1:** The basic clinical characteristics of the object of study

	Case 1	Case 2	Case 3	Case 4	Case 5	Con 1	Con 2
Year (2015)	Death	56	54	52	17	45	21
Sex	Female	Female	Female	Female	Male	Male	Female
Year of initial diagnosis	1990	2006	2007	1992	2014	−	−
Smoking	−	−	−	−	−	−	−
Drinking	−	−	−	−	−	−	−
**Clinical manifestation**							
Heart	+	+	+	+	+	−	−
Proptosis	+	+	−	+	+	−	−
Hands tremble	+	+	+	+	+	−	−
Goiter	−	−	+	+	+	−	−
Ophthalmopathy	−	−	−	+	−	−	−
**Serological test**							
FT3 (pmol/L)	3.26	4.76	6.86	2.39	9.85	4.49	4.70
FT4 (pmol/L)	16.57	17.60	21.61	7.71	26.50	16.45	15.94
TSH (mUL/L)	< 0.005	0.118	< 0.005	62.10	0.072	1.18	0.740
TRAb (IU/L)	−	2.38	4.13	−	5.75	1.27	1.04
Treatment	Thiamazole	Thiamazole	Thiamazole	I^131^	Thiamazole	−	−

+: positive; −: negative

### Thyroid function examination and susceptibility gene screening

Detection of thyroid hormones and TRAb by chemiluminescence method(Cobase411) was used and the reference range of indicators are: FT3: 2.3–6.8 pmol/L; FT4: 10–23.5 pmol/L; TSH: 0.34 ~ 4.0mIU/L; TRAb: 0 ~ 1.75 IU/L. For preparation of DNA, genomic DNA from EDTA-treated peripheral blood was extracted according to DNA extraction kit manual (Tiangen Biochemical Technology Co., Ltd.)

The Agilent SureSelect Human All Exome Kit(Agilent) was used for exon capture. The sequencing processes were exon capture, hybrid library cleaning and purification, PCR amplification of exon DNA library, library quality detection, sequencing, and data analysis. The PCR reaction conditions of PCR Amplification was initially 30 s at 98 °C, 10 cycles of 98 °C for 10 s, 10 cycles of 60 °C for 30 s, 10 cycles of 72 °C for 30 s, 10 cycles of 72 °C for 5 min at 72 °C, followed by a final extension of 4 °C. The exome region was sequenced by illumine hiseq2500, and GATK standard procedure was adopted to calibrate the initial data. Quality control of raw reads was performed with fastqc. Transition and Transversion (SNV) and Insertion and Deletion (InDel) of each sample were detected though VarScan and GATK HaplotypeCaller. In addition, SNV stands for single nucleotide variants and SNP stands for single nucleotide polymorphism. Both concepts refer to single nucleotide changes, but SNPs are generally two-state and SNV has no such restriction. In addition, if the frequency of the single-base variation in a species reaches a certain level, it is called SNP, and if the frequency is unknown (for example, only found in an individual), it is called SNV. Sanger sequencing was used to confirm the genotype variant of 6 genes (*MAP7D2*, *SLC1A7*, *TRAF3IP3*, *PTPRB*, *PIK3R3*, *DISC1*) that were in all the participants.

## Results

### The quality of raw data

The sequencing quality Q value was used to evaluate the sequencing error rate of the base. The base quality value Q20 indicated that the error rate was 1%. Similarly, the base quality value Q30 indicated that the error rate was 0.1%. In the case study, the data revealed that the sequencing quality of the seven samples were high (Table 2).

**Table 2:** Raw data sequencing data statistics

	R1				R2				Mean Bait Coverage (fold)
	Total Reads	Reads length	Q20 (%)	Q30 (%)	Total Reads	Reads length	Q20 (%)	Q30 (%)	
Case1	59,415,807	35–151	99.98	97.51	59,415,807	35–151	99.6	92.65	144.47
Case2	43,894,852	35–151	99.98	97.60	43,894,852	35–151	99.67	93.46	112.48
Case3	71,415,598	35–151	99.97	97.61	71,415,598	35–151	99.64	93.18	143.06
Case4	74,526,096	35–151	99.98	97.58	74,526,096	35–151	99.64	93.41	200.99
Case5	80,885,417	35–151	99.98	97.57	80,885,417	35–151	99.62	93.09	211.79
Con1	80,289,875	35–151	99.98	97.55	80,289,875	35–151	99.47	92.06	207.86
Con2	69,913,521	35–151	99.98	97.50	69,913,521	35–151	99.66	93.88	155.40

"Q20 (%)" and "Q30 (%)" respectively indicate the ratio that the sequencing quality value Q in the raw data is not lower than Q20 and Q30

The base average coverage depth of all samples was larger than 100 × which meant that the detected SNV was reliable.

### SNV/InDel detection and annotation of 7 samples

The SNV/InDel locus that was discovered with both VarScan and GATK methods was to be of high quality. If they were not discovered by both VarScan and GATK methods, the locus was medium quality. There were 144,169 high quality SNV/InDel locus involved in this study (Table 3).

**Table 3:** The SNV/InDe locus number statistics in 7 samples

Category	SNV(N)	InDel(N)
Both	125,581	18,588
Only Varscan	51,959	12,118
Only GATK	5337	2250

**Fig. 2:**
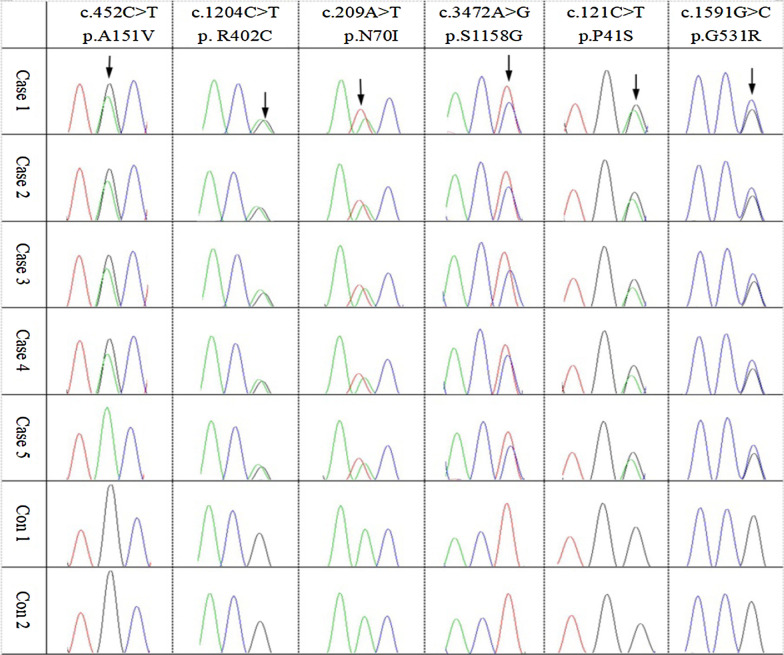
The Sanger sequencing of variations for the 7 participants

**Fig. 3:**
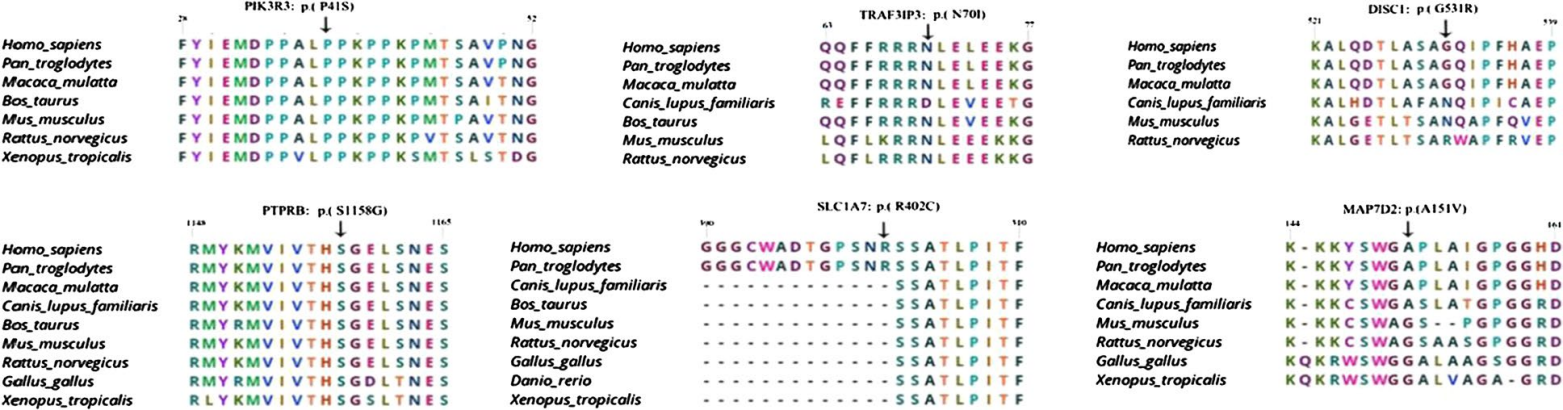
Amino acids conservation in orthologous species for the variants

The variations carried by the patient were identified as susceptibility genes of GD in the three-generation family, including *MAP7D2*(c. 452C > T: p. A151V), *SLC1A7*(c. 1204C > T: p. R402C), *TRAF3IP3*(c. 209A > T: p. N70I), *PTPRB*(c. 3472A > G: p. S1158G), *PIK3R3*(c. 121C > T: p. P41S), *DISC1*(c. 1591G > C: p. G531R). All SNV/InDel sites in the case study were rare variants according to several known population databases (Freq_Alt1000, Kaviar_20150923, ESP6500, gnomAD). In addition, Chinese Millionome Database (CMDB, https://db.cngb.org/cmdb/) were applied for the comparative analysis. CMDB contain considerable variation and their allele frequency information came from 141,431 unrelated healthy Chinese individuals (Phase I results). SNV function analysis indicated that all the SNV of *MAP7D2*, *SLC1A7*, *TRAF3IP3*, *PTPRB*, *PIK3R3*, *DISC1* were of nonsynonymous variations. The genotype of the 6 genetic variations from the 7 samples by Sanger sequencing was the same with the results from the whole exome sequencing (Fig. 2). The PCR premiers of six genetic variants were shown in Table 4. Based on PolyPhen-2 prediction and the amino acids conservation analysis in orthologous species, the rs555004337 in *TRAF3IP3*, rs186466118 in *PTPRB*, and rs115181807 in *PIK3R3* were likely to affect the protein function (Table 5 and Fig. 3). These genes involved in the Biological Process, Molecular Function, Cellular Component, and KEGG pathway were showed in an Additional file 1.

**Table 4:** The PCR primers of six genetic variants

Gene: variant	Premiers
*MAP7D2:* c. 452C > T	F-ATCTGAAAGTGGTGCCTCTGAA R-TAGCCTAGCCGCATTGTTTACT
*SLC1A7:* c. 1204C > T	F-GTTGACCTGGGCGATGAAGA R-AAACACCTCCCTCATAGGAAGAAC
*TRAF3IP3:* c. 209A > T	F-AACAGGTGCTTGGAGGTCATC R-AGCACACAGCAGTATGTCCCTT
*PTPRB:* c. 3472A > G	F-GGAAACTAAGGACCAACCAAGG R-CACTGCATTTCCCTCCCTCA
*PIK3R3:* c. 121C > T	F-GCATTCTAGTTACCTTGAAATATCC R-CATACCTTGGTTAGTGAGCTGCT
*DISC1:* c. 1591G > C	F-GGAAATAGAGGAGCAAGAGCAG R-CAGACTGCTTGGGAAATGTTTAG

**Table 5:** The details of SNV/InDel information

Gene	*MAP7D2*	*SLC1A7*	*TRAF3IP3*	*PTPRB*	*PIK3R3*	*DISC1*
**Information**						
Position	X:20,074,820	1:53,556,483	1:209,933,593	12:70,956,666	1:46,546,408	1:231,906,773
exon	4	8	3	14	2	6
SNP ID	rs750367268	–	rs555004337	rs186466118	rs115181807	rs56229136
Ref allele	G	G	A	T	G	G
Alt allele	A	A	T	C	A	C
Function	missense	missense	missense	missense	missense	missense
PolyPhen-2 score	0.139(benign)	–	0.729(possibly damaging)	0.521(possibly damaging)	1(probably damaging)	0.059(benign)
Freq_Alt (1000)	–	0.000199	0.001797	0.004193	–	0.000599
Kaviar_ 20,150,923	0.0000194	0.0000388	0.0006015	0.0015006	–	0.0003428
ESP6500	–	–	–	–	–	0.000077
gnomAD	0.000011	0.000028	0.0006	0.0019	0.000022	0.0004
CMDB	–	–	–	0.0038	0.0051	–
**Genotype**						
Case 1	G/A	G/A	A/T	T/C	G/A	G/C
Case 2	G/A	G/A	A/T	T/C	G/A	G/C
Case 3	G/A	G/A	A/T	T/C	G/A	G/C
Case 4	G/A	G/A	A/T	T/C	G/A	G/C
Case 5	A/A	G/A	A/T	T/C	G/A	G/C
Con 1	G/G	G/G	A/A	T/T	G/G	G/G
Con 2	G/G	G/G	A/A	T/T	G/G	G/G

CMDB: Chinese Millionome Database; –: No found

## Discussion

GD is an autoimmune disease with complex etiology. With the extensive development of GWAS research, many GD susceptibility genes have been identified such as HLA, CTLA4, PTPN22, and TSHR [9]. Gene mutations may affect the antigen presentation, T cell signal transduction, B cell antibody production, thyroid hormone, and thyroid-related apoptosis which may lead to the occurrence of GD. The gene mutation effects provide a theoretical basis for GD's precise diagnosis and treatment. However, the current impact of GD susceptibility gene polymorphism on the expression of corresponding proteins is still unknown and research on the interaction between genes is limited in elucidating the role of gene polymorphism in disease pathogenesis.

In the case study, it presents a rare familial GD case in 5 patients in a three-generation family. The five patients are consistent with the general characteristic of GD patients which is that the GD is prone to attack women at the age of 30–60 [10]. All the members of the three-generation family came from the same district of Zhanjiang city and they have been living in similar environment which guarantees the consistency of environmental factors in this case study. Although the etiology of GD is complex and clear identification of potential factors for GD has not been completed, it is widely recognized that the genetic determinants such as *HLA*, *CTLA-4*, *PTPN22*, and *CD40* have contributed to the risk of GD [11]**.** However, no variation of these former identified genes was found, but the following variations of *MAP7D2*, *SLC1A7*, *TRAF3IP3*, *PTPRB*, *PIK3R3*, *DISC1*, and SUPT20HL were found in the familial GD. Furthermore, the variations in *PTPRB*, *PIK3R3*, and *TRAF3IP3* were predicted to have alter the functions of the encoded protein. Familial GD of multigeneration is important for heritable studies because it avoids the genetic heterogeneity factor, so this case study may explain the genetic cause of the familial clustering of GD.

*MAP7D2* (MAP7 domain containing 2) is located on X chromosome. *MAP7D2* is specifically expressed in human brain tissue which has impact on the behavioral traits and cognition in human. *MAP7D2* is also associated with sex-biased mental illnesses [12]. Previous studies has shown that gender predisposition to GD is associated with X chromosome inactivation (XCI) migration [13]. Thus, the *MAP7D2* study is likely to provide important general information about the reason why women are more vulnerable to GD. *SLC1A7* and *DISC1* are also susceptibility genes for mental illnesses. In one research, Keith A. Young et al. discovered that *DISC1* gene played a vital role in post-traumatic stress disorder (PTSD) severity of US military veterans [14]. In another research, Fujita K, et al. revealed that *SLC1A7* gene expression in peripheral blood leukocytes was responsible for the association between socioeconomic status and depressive mood in healthy adults [15]. We speculated that *SLC1A7* and *DISC1* are involved in regulating the symptoms of GD such as nervousness and irritability

The protein encoded by *PTPRB* belongs to the family of protein tyrosine phosphatases (PTP). The activation of PTK (protein tyrosine kinase) was regulated through the binding of SH2 domains from PI3K(*PIK3R3* gene encode). The balance of tyrosine protein phosphorylation was regulated by PTP/PTK which participated in cell signal transduction, cell growth regulation, differentiation, metabolism, transcription, immune responses, etc. Researches have demonstrated that the significant role of PTKs and PTPs were to modulate the tyrosine phosphorylation-dependent signaling pathways which were critical for the effector of NK cell and Neutrophil cell [16, 17]. *TRAF3IP3* is also highly expressed in CD34 + CD38 + CD7 + common lymphoid progenitors (CLPs) Furthermore, CD34 + CD38 + CD7 + cells have the capacity to differentiate into B/NK/T cell which implies that *TRAF3IP3* possibly may play a role in lymphoid development [18]. The overactivation of the T/B cell was regulated by *CTLA-4* and *CD40* gene variants which has been confirmed in the pathogenesis of autoimmune diseases including GD. Therefore, further studies are necessary to figure out whether the variations of *PTPRB*, *PIK3R3* and *TRAF3IP3* are involved in the dysfunction of thyroid autoimmune.

The case study is about the susceptibility genes of a single three-generation family of Graves disease, but there are insufficient samples of similar families to verify the results of this study. Also, the frequency of susceptibility genes screened in this study has not been further verified in the population of patients with sporadic Graves disease and the relationship between these gene mutations and sporadic Graves disease is uncertain. Finally, the susceptibility genes screened this time need to be further studied at the protein molecular level to further determine the biological significance of these mutations.

## Conclusion

To summarize, the WES was applied to establish an association between *MAP7D2*, *SLC1A7*, *TRAF3IP3*, *PTPRB*, *PIK3R3*, *DISC1* genes and the familial GD of a three-generation family. The findings in this cast studies are clues for further study and more verification and function researches are needed to explore these genes related to GD susceptibility.

## Supplementary information

**Additional file 1**. Gene function analysis.

## Data Availability

The datasets analysed during the current study are available in the Genome Sequence Archive for human repository (https://bigd.big.ac.cn/gsa-human/), under the accession code: HRA000505.

## Abbreviations

GD: Graves’ disease
WE: Whole exome sequencing
